# Exploitation of Potentially New Antibiotics from Mangrove Actinobacteria in Maowei Sea by Combination of Multiple Discovery Strategies

**DOI:** 10.3390/antibiotics8040236

**Published:** 2019-11-27

**Authors:** Qin-Pei Lu, Jing-Jing Ye, Yong-Mei Huang, Di Liu, Li-Fang Liu, Kun Dong, Elizaveta A. Razumova, Ilya A. Osterman, Petr V. Sergiev, Olga A. Dontsova, Shu-Han Jia, Da-Lin Huang, Cheng-Hang Sun

**Affiliations:** 1 Department of Microbial Chemistry, Institute of Medicinal Biotechnology, Chinese Academy of Medical Sciences & Peking Union Medical College, Beijing 100050, China; qinpei89@hotmail.com (Q.-P.L.); LiuLiFang@imb.pumc.edu.cn (L.-F.L.); 2 Beijing Key Laboratory of Antimicrobial Agents, Institute of Medicinal Biotechnology, Chinese Academy of Medical Sciences & Peking Union Medical College, Beijing 100050, China; 3 College of Basic Medical Sciences, Guilin Medical University, Guilin 541004, China; yejingjing@stu.glmc.edu.cn (J.-J.Y.); jsh@stu.glmc.edu.cn (S.-H.J.); 4 Zhanjiang for R&D Marine Microbial Resources in the Beibu Gulf Rim, Marine Biomedical Research Institute, Guangdong Medical University, Zhanjiang 524023, China; huangym@gdmu.edu.cn; 5 College of Life Sciences, Jiamusi University, Jiamusi 154007, China; liudi96@hotmail.com; 6 College of Life Science and Technology, China Pharmaceutical University, Nanjing 211198, China; Kun.Donglhr@outlook.com; 7 Department of Chemistry, Lomonosov Moscow State University, Moscow 119992, Russia; elizaveta_razumova@list.ru (E.A.R.); osterman@yandex.ru (I.A.O.); petya@genebee.msu.ru (P.V.S.); dontsova@genebee.msu.su (O.A.D.); 8 Center of Life Sciences, Skolkovo Institute of Science and Technology, Moscow 143025, Russia; 9 Shemyakin-Ovchinnikov Institute of Bioorganic Chemistry, Russian Academy of Sciences, Moscow 119992, Russia

**Keywords:** Maowei Sea, mangrove, actinobacteria, diversity, ESKAPE, dereplication, GNPS, quinomycins

## Abstract

Rediscovery of known antibiotics from actinobacteria, especially *Streptomyces*, has become a bottleneck issue. Nowadays, more specific identification and dereplication could be acquired by a combination of modern analytic techniques with various databases. In this study, 261 actinobacterial strains were isolated from 8 mangrove soil samples by culture-dependent method. A total of 83 strains were selected to evaluate antibacterial activities and mechanisms by disc diffusion method and a unique double fluorescent protein reporter system (pDualrep2), respectively. Thirty-two strains exhibited antagonistic activity against at least one of the “ESKAPE” pathogens. Four *Streptomyces* strains (B475, B486, B353, and B98) showed strong inhibitory activity against Gram-positive bacteria and induced DNA damage SOS response. One *Micromonospora* strain (B704) exhibited inhibitory activity against several pathogens and induced attenuation-based translational inhibitors reporter. Seven members of quinoxaline-type antibiotics including quinomycin A, quinomycin monosulfoxide, and other five putative new analogues were found from the culture broth of strain B475 by a combination of anti-MRSA guide, HPTLC, HPLC-UV, and UPLC-UV-HRESIMS/MS analysis, Chemspider searching, and MS/MS-based molecular networking analysis. In conclusion, this study not only demonstrated that mangrove is a rich source of actinobacteria with the potentially new antibiotics but showed rapid dereplication of known antibiotics in the early stage can improve efficiency for the discovery of new antibiotics.

## 1. Introduction

The increasing prevalence of antibiotic-resistant bacteria, along with the rapid development of cross-resistance with antibiotics worldwide, is one of the most serious threats to public health in the 21st century. All classes of antibiotics have seen the emergence of resistance compromising their use. Hence, new antibiotics are urgently needed to combat effectively against antibiotic-resistant bacteria, especially the “ESKAPE” pathogens (*Enterococcus faecium*, *Staphylococcus aureus*, *Klebsiella pneumoniae*, *Acinetobacter baumannii*, *Pseudomonas aeruginosa,* and *Enterobacteriaceae*) [1,2,3,4].

Antibiotic discovery efforts can be divided into two streams with distinct origins: natural products and synthetic antibacterials [5]. Despite the chemically synthetic efforts, natural environments are still the leading source for the discovery of novel antibiotics [6,7]. As for natural products, actinobacteria produce approximately two-thirds of all known antibiotics, the majority of which are produced by *Streptomycetes*. Consequently, these microorganisms are significant in the fight against emerging multidrug-resistant pathogens [8]. Historically, the intensive exploration of terrestrial actinobacteria has yielded many important drug leads, later developed into antibiotics used in the clinic (such as streptomycin, erythromycin, etc.). Meanwhile, the frequent rediscovery of known antibiotics became a bottleneck issue, as Kim Lewis mentioned “the more we know about antibiotics, the fewer we can discover” [9,10]. Nowadays, it is much more challenging than before to discover new antibiotics by simply using actinobacteria-based screening [11,12]. Thus, many new strategies and innovative approaches are involved to break through the issue, such as exploitation of new antibiotics from unique and underexplored habits, novel culturing approaches to isolate novel species thought to be uncultivable, new screening models, activation of cryptic biosynthetic pathway, genomes mining [7,13,14,15,16,17,18], and using modern analytic techniques coupled with powerful databases [19,20,21,22,23,24,25], since it is believed that actinobacteria is still a source of novel antibiotics [26,27].

As one of the world’s most dynamic environments, mangrove forests are a unique and little exploited ecosystem for actinobacteria. The wetlands are distributed in the inter-tidal estuarine zones in the tropical and subtropical regions of the world and represent a rich biological diversity of plants, animals, and microorganisms [28,29,30]. The mangrove environment’s elements, such as geographical location, pH, temperature, salinity, moisture, and nutrients, differ significantly in different regions so that mangrove actinomycetes are diverse and unique [31]. In the recent decade, studies on mangrove derived actinomycetes and their secondary metabolites have become a hotspot to discover new antibiotics. To date, at least 88 new actinobacterial species, including eight novel genera, have been isolated from mangrove, and more than 80 new compounds including some promising molecules such as salinosporamide A, halichoblelide D, xiamycins, and indolocarbazoles have been reported from mangrove-derived actinobacteria [27,32,33,34,35,36,37,38].

In this study, we aimed to investigate the diversity, antibacterial activity, and potential to produce new bioactive secondary metabolites of culturable actinomycetes isolated from the Maowei Sea Mangrove Reserve, a relatively unexplored region in Qinzhou City, Guangxi Zhuang Autonomous Region, China. Culture-dependent approaches, utilizing a variety of media, were employed to select for Actinomyces. Their ability to produce antimicrobial activity against “ESKAPE” pathogens was evaluated by disc diffusion method. Meanwhile, a high-throughput screening model based on the double fluorescent protein reporter was also implemented to find the strains producing secondary metabolites as ribosome or/and DNA biosynthesis inhibitors. At last, the secondary metabolites of the *Streptomyces* sp. B475 were prioritized by analysis with comprehensive approaches, including bioassay-guided fractionation, hyphenated technique, database identification, and MS/MS-based molecular networking analysis.

## 2. Results

### 2.1. Isolation and Diversity of Actinobacteria from Mangrove Soil of Maowei Sea

**Figure 1:**
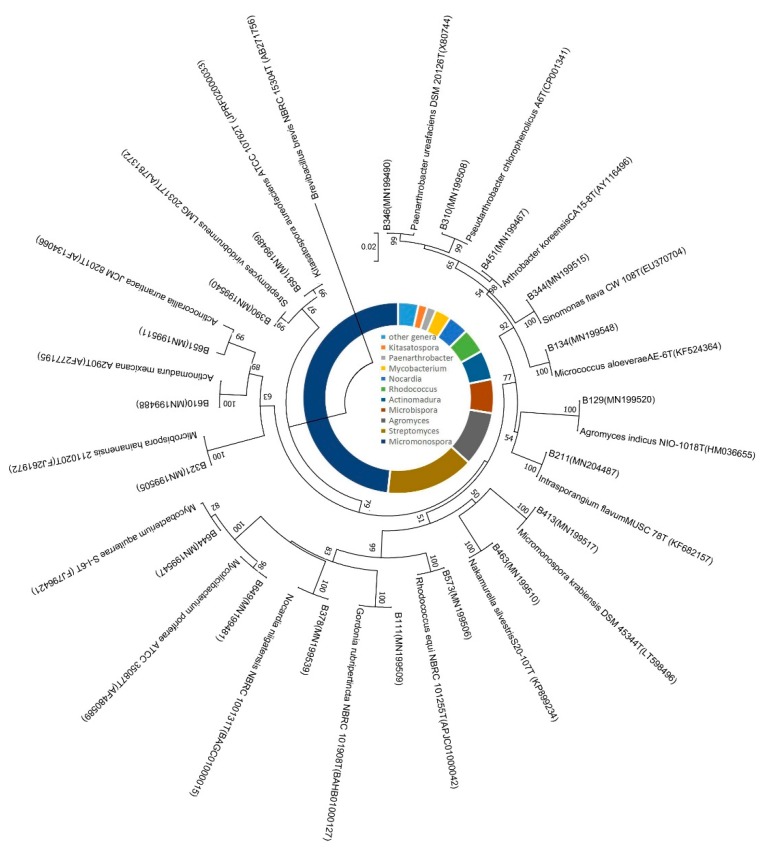
Phylogenetic tree based on the 16S rRNA gene sequences (>750 bp) using neighbor-joining method for 19 representative actinobacterial strains and their closely related type strains. Numbers at nodes indicate the level of bootstrap support (>50%) based on 1000 replications. *Brevibacillus brevis* was used as an outgroup. Bar, 2 nt substitutions per 100 nt.

**Figure 2:**
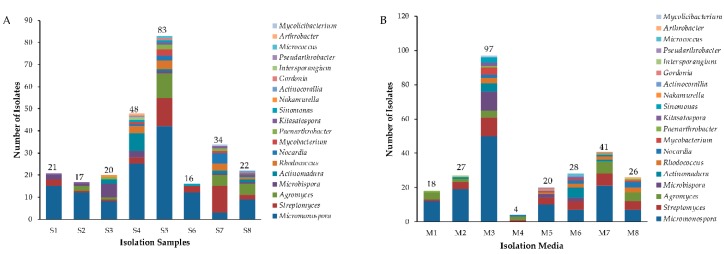
Diversity of culturable actinobacteria from mangrove soils of the Maowei Sea. (**A**) The number of actinobacterial isolates recovered from the different samples of mangrove soils. (**B**) The number of actinobacterial isolates from different culture media.

In total, 750 strains were isolated and purified from 8 mangrove soil samples by using eight different isolation media (Table S1). Among them, 261 isolates were identified as actinobacterial strains by partial 16S rRNA gene sequence (>750 bp) comparison and further assigned to 19 genera in 10 families of 6 orders (Figure 1). The predominant genus was *Micromonospora* (48.0 %, 126 strains) followed by *Streptomyces* (15.0 %, 38 strains), the others were *Agromyces* (24 strains), *Microbispora* (15 strains)*, Actinomadura* (13 strains), *Rhodococcus* (11 strains), *Nocardia* (9 strains), *Mycobacterium* (6 strains), *Kitasatospora* (4 strains)*, Paenarthrobacter* (4 strains), *Sinomonas* (3 strains), *Pseudarthrobacter* (1 strain)*, Micrococcus* (1 strain), *Intersporangium* (1 strain), *Gordonia* (1 strain), *Nakamurella* (1 strain), *Actinocorallia* (1 strain)*, Arthrobacter* (1 strain)*,* and *Mycolicibacterium* (1 strain). The genera distribution of the 261 actinobacterial strains is listed in Table 1, and their distribution in 8 mangrove soil samples is shown in Figure 2A and Table S2. Sample 5 exhibited the highest diversity (13 genera), followed closely by both sample 4 and sample 7 (11 genera), sample 8 (9 genera), sample 3 (7 genera), sample 2 (5 genera), sample 1 (4 genera) and sample 6 (3 genera). Among the 8 isolation media used, M3 media proved to be most successful in terms of diversity and number of isolated actinobacterial strains; totally, 97 actinobacterial strains distributed in 12 genera were obtained. M6 produced the second-highest diversity of isolates (28 strains in 10 genera), followed by M7 (41 strains in 8 genera) and M8 (26 strains in 8 genera). However, M4 yielded the lowest number and diversity of isolates (4 strains in 3 genera) (Figure 2B and Table S3).

### 2.2. Antibacterial Assay and Mechanism Determination

Out of 261 actinobacterial strains, 83 strains were selected to evaluate antibacterial activities against six sets of indicator bacteria, including drug-sensitive and drug-resistant “ESKAPE” strains by the traditional disc diffusion method. Meanwhile, for inspection of their underlying antibacterial mechanism, a unique double fluorescent protein reporter system (pDualrep2) was used.

Thirty-two strains exhibited antagonistic activity against at least one of the tested bacteria, they distributed in 8 genera including *Micromonospora* (14), *Streptomyces* (10), *Agromyces* (3), *Nocardia* (1), *Kitasatospora* (1), *Intrasporangium* (1), *Nakamurella* (1), and *Mycolicibacterium* (1) (Table 1 and Table S4). The number of strains active against Gram-negative bacteria was lower than the number of strains active against Gram-positive bacteria. A total of 15 strains were active against at least one of the Gram-negative test strains while 27 strains were active against at least one of the Gram-positive test strains. Among them, ten strains showed inhibitory activities against both Gram-positive and Gram-negative bacteria.

**Figure 3:**
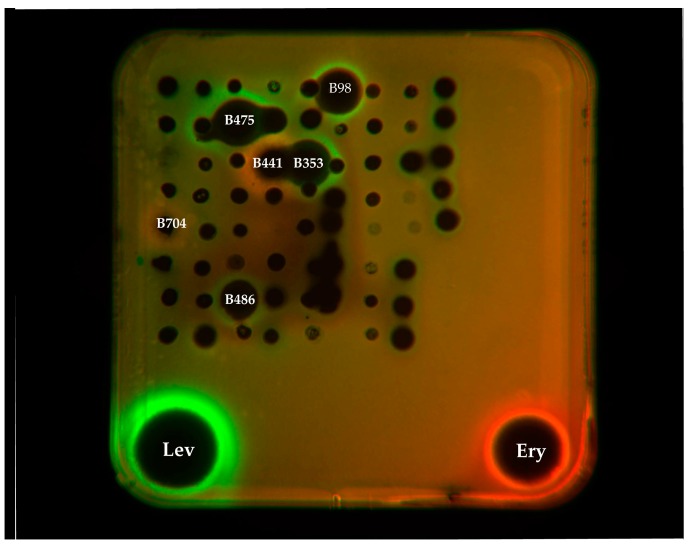
Induction of a double fluorescent protein reporter system sensitive to inhibitors of the ribosome progression or inhibitors of DNA replication, respectively. Spots of erythromycin (Ery), levofloxacin (Lev), and tested samples were placed on the surface of an agar plate containing *E. coli* tolC cells transformed with the pDualrep2 reporter plasmid. Shown is the fluorescence of the lawn of *E. coli* cells scanned at 553/574 nm (green pseudocolor) for RFP fluorescence and 588/633 nm (red pseudocolor) for Katushka2S fluorescence. RFP is upregulated by induction of DNA damage SOS response, while the induction of expression of Katushka2S is triggered by translation inhibitors.

Ethyl acetate (EA) extracts from the culture broths of 83 strains were screened by a double fluorescent protein reporter system (pDualrep2). Both strain B441 (S*treptomyces*) and B704 (*Micromonospora*) could induce Katushka2S expression, acting as a typical translation inhibitor of ribosome as erythromycin did. Meanwhile, four *Streptomyces* strains (B475, B486, B353, and B98) could induce DNA damage SOS response, acting as a typical inhibitor of topoisomerase like levofloxacin did (Figure 3).

### 2.3. Identification and Dereplication of Antibacterial Components Produced by Strain B475

**Figure 4:**
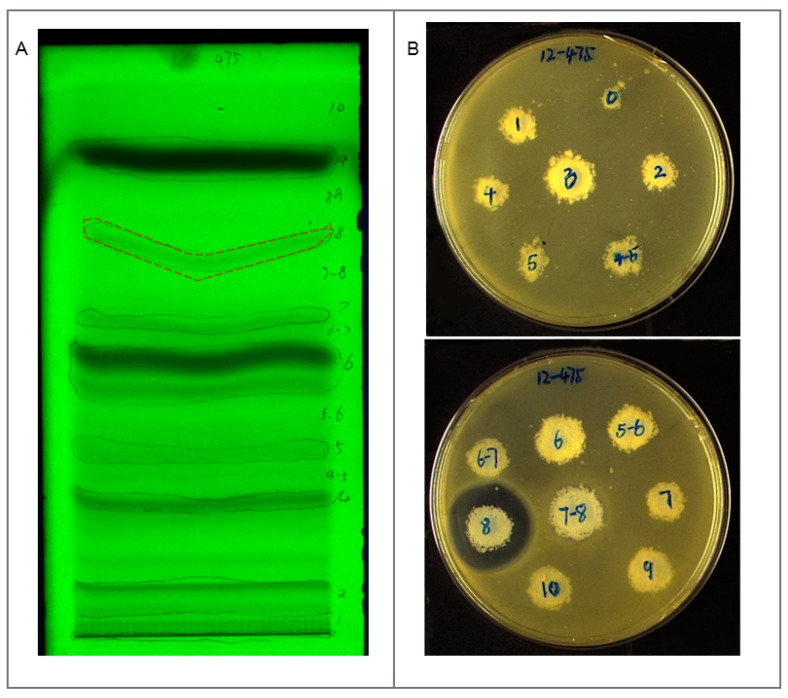
TLC profile of the Ethyl acetate (EA) extract of strain B475 and anti-methicillin-resistant *Staphylococcus aureus* (MRSA) results of each band. (**A**) TLC profile of EA extract of strain B475 at UV 254 nm, band 8 (red). (**B**) The anti-MRSA results of the bands from TLC; only band 8 showed inhibition zone.

**Figure 5:**
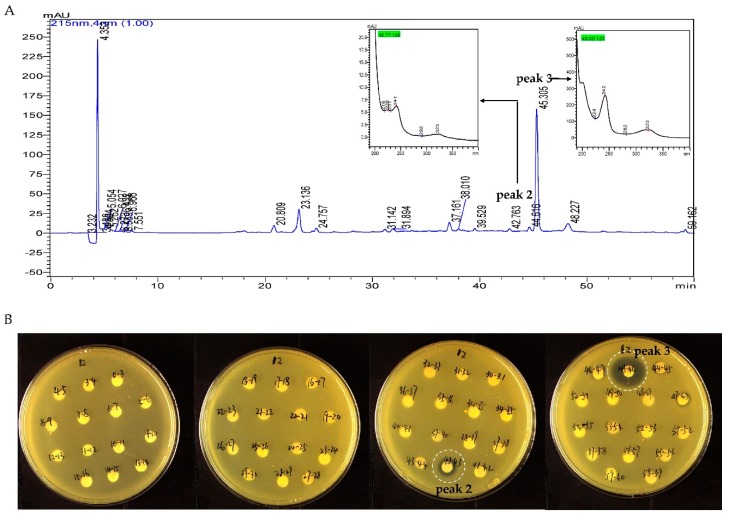
HPLC profile of band 8 and anti-MRSA results of each fraction. (**A**) The HPLC profile of band 8 from EA extract of strain B475; UV spectrum in the left: the active peak at R_t_ = 42.76 min; UV spectrum in the right: the active peak at R_t_ = 45.30 min. (**B**) The anti-MRSA results of each fraction collected from HPLC, two fractions collected from 42–43 min and 45–46 min showed inhibition zones; the diameters of inhibition zones were 10 mm and 16 mm, respectively.

EA extract of strain B475 was directed to TLC and separated by mobile phase with Dichloromethane: Methanol = 10:1 (v/v), 15 bands in the TLC plate could be observed under UV at 254 nm (Figure 4A). The antibacterial assay of these bands against methicillin-resistant *Staphylococcus aureus* (MRSA) showed the only band 8 was active (Figure 4B). The band 8 was further separated by HPLC. Based on time-dependent fractionation, a total of 58 fractions were collected, dried under vacuum, and dissolved in methanol to screen against MRSA strains using the disc diffusion method. Two fractions with retention time (R_t_) 42–43 min and 45–46 min exhibited the apparent inhibitory zone with a diameter of 10 mm and 16 mm respectively (Figure 5). The active fractions were further analyzed by UPLC-UV-HRESIMS/MS (Waters Xevo G2-XS QTof).

**Figure 6:**
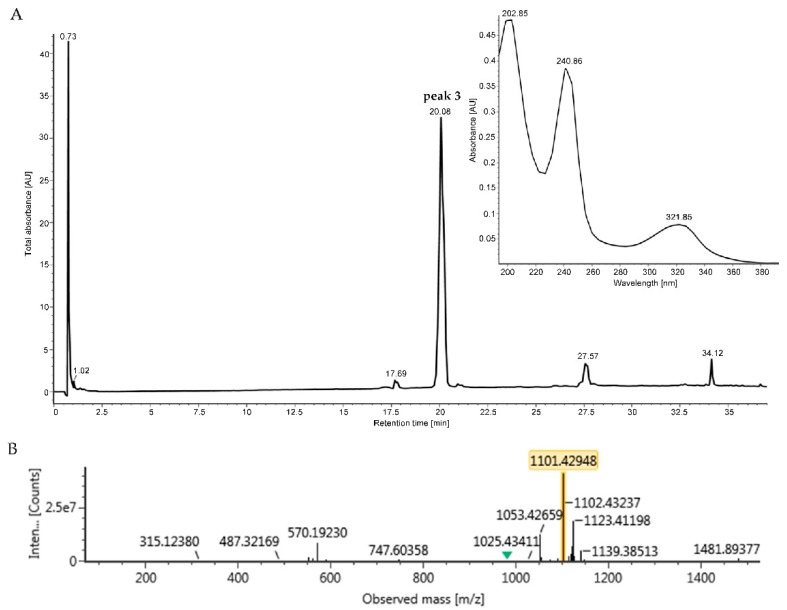
The UPLC-UV-HRMS/MS analysis and dereplication of peak **3**. (**A**) UPLC profile of peak **3** at R_t_ = 20.06 min and its UV spectra. (**B**) Mass spectra of peak **3**.

The peak at R_t_ = 45–46 min from HPLC was identified as peak **3** at R_t_ = 20.08 min in UPLC-UV-HRESIMS/MS chromatogram, which had the monoisotopic weight of 1101.4295 [M+H]^+^ and 1123.4120 [M+Na]^+^ and characteristic absorption wavelength of UV at 243 nm and 321 nm (Figure 6). This spectral data of peak **3** was used to search in the database of Chemspider coupled with Waters UNIFI software and hit the molecular weight of echinomycin, also known as quinomycin A, which also showed the similar UV absorption with peak **3**. Therefore, peak **3** was identical to the echinomycin, which was further confirmed by comparison of the ^1^H NMR spectrum of peak **3** with that of echinomycin [39,40] (Figure S1). A similar approach, another bioactive peak at R_t_ = 42–43 min, showing similar UV absorption of peak **3** in HPLC, was identified as peak **2** at R_t_ = 18.79 min with *m/z* at 1055.4421 [M+H]^+^ in UPLC-UV-HRESIMS/MS chromatogram (Figure S2). Based on data of peak **2**, four hits were retrieved when searching in the Chemspider database, but none of their UV spectra matched with those.

**Figure 7:**
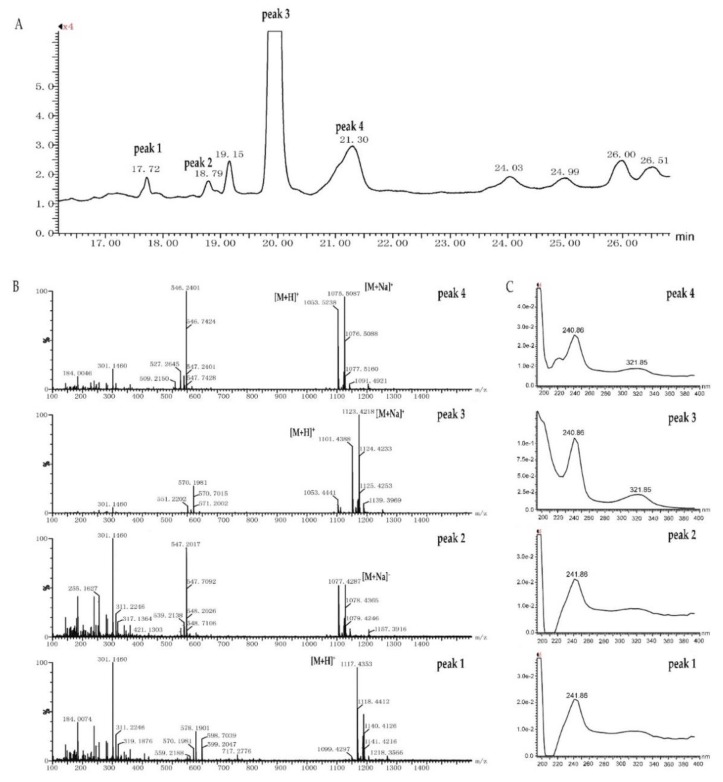
UPLC-UV-MS chromatogram of the sample eluted from band 8 in the TLC plate. (**A**) UPLC profile of the sample eluted from band 8 in the TLC plate, quinomycin monosulfoxide (peak **1**), echinomycin (peak **3**), two putative new analogs (peak **2**, peak **4**). (**B**) The mass spectra of quinomycin monosulfoxide (peak **1**), echinomycin (peak **3**), peak **2**, and peak **4**. (**C**) UV spectra of quinomycin monosulfoxide (peak **1**), echinomycin (peak **3**), peak **2**, and peak **4**.

For the discovery of potentially new quinoxaline-type antibiotics, the sample eluted from band 8 in the TLC plate was directly analyzed by UPLC-UV-HRESIMS/MS. Besides peak **2** and **3**, another two small peaks (peak **1**, R_t_ = 17.72 min, *m/z* at 1117.4353 [M+H]^+^; peak **4**, R_t_ = 21.30 min, *m/z* at 1053.5238 [M+H]^+^) shared the similar UV absorption of peak **3**, suggesting that these two peaks were analogs of echinomycin as well (Figure 7). Closer examination of data against Chemspider database and literature indicated that peak **1** was quinomycin monosulfoxide, and peaks **2** and **4** were potentially new members of quinoxaline-type antibiotics (Table S5).

### 2.4. MS/MS Network-based Discovery of Potentially New Antibiotics Produced by Strain B475

**Figure 8:**
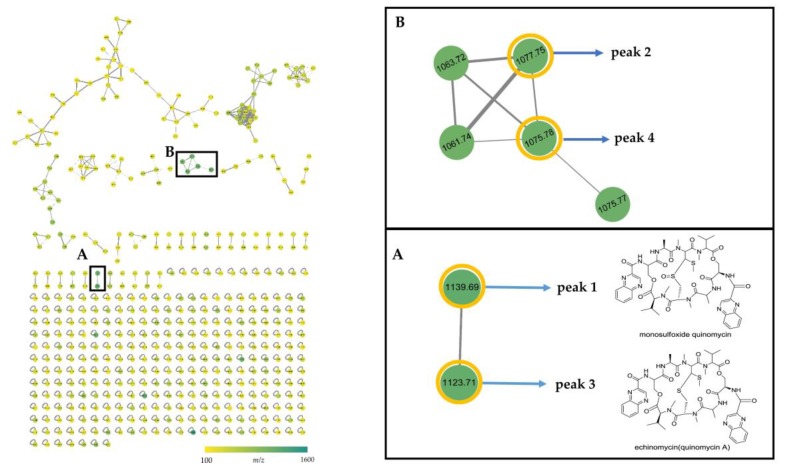
The molecular network generated by the MS/MS data from the EA extract of strain B475. Two clusters were related to the quinoxaline antibiotics (**A**,**B**).

In order to explore new analogues of quinoxaline antibiotics in EA extract of strain B475, UPLC-HRESIMS/MS-based dereplication analysis was performed by using the GNPS web platform [22]. The fragmentation data were organized by molecular networking and subsequently dereplicated against spectra in the GNPS database. In the molecular network, the spectra were organized into 472 nodes with 528 edges forming 40 clusters of connected nodes (≥2 nodes) and 295 individual nodes, and the nodes were represented by precursor mass with a different color (Figure 8). No hits against the GNPS database were found in the crude extract of strain B475. However, two clusters (cluster A and B) appeared and were of particular interest due to the presence of precursor mass matching with the quinoxaline antibiotics (peak **1**–**4**). In cluster A, the structurally related quinoxaline A (peak **3**, *m/z* 1123.71, [M+Na]^+^) and quinomycin monosulfoxide (peak **1**, *m/z* 1139.69, [M+Na]^+^) were directly connected to each other, and the mass difference of 15.98 between two nodes corresponded to the neutral loss of oxygen. In the cluster B, two new putative quinoxalines, peak **2** (*m/z* 1077.75, [M+Na]^+^) and peak **4** (*m/z* 1075.78, [M+Na]^+^), were connected to three additional nodes (*m/z* 1063.72, 1061.74, and 1075.77), suggesting the presence of other three putative quinoxalines analogues in the extract. Compared with the molecular weight of the known quinoxalines, these five components in cluster B were potentially new members of quinoxalines.

## 3. Discussion

Actinobacteria are widely dispersed throughout the mangrove environments [34,37,38,41,42,43,44,45,46]. In this study, to investigate actinobacterial biodiversity and harvest more potentially antibacterial strains, eight mangrove soil samples from different sites of Maowei Sea Mangrove Reserve were collected. A total of 261 actinobacterial strains in 19 genera from 10 families of 6 orders were isolated from eight different media. The results not only demonstrated the rich actinobacterial diversity in Maowei Sea Mangrove Reserve but also provided many strains of different genus including rare actinomycetes for assay.

So-called “ESKAPE” pathogens were originally coined by the infectious Diseases Society of America (IDSA) to emphasize their capacity to “escape” from conventional antibacterial treatment. Thus, this group of drug-resistant pathogenic bacteria receives the most attention in drug discovery and screening programs [3,47]. In this study, 12 strains including drug-sensitive and drug-resistant “ESKAPE” pathogens were used as indicator strains. Out of 83 selected strains, 12 strains had strong inhibitory activity (inhibition zone >20 mm) against Gram-positive bacteria. This group of 12 strains contained seven *Streptomyces* strains (B486, B290, B448, B475, B390, B353, B98), four *Micromonospora* strains (B42, B331, B409, B277), and one *Nocardia* strain (B391). Among them, strain B42 also showed strong inhibitory activity against Gram-negative bacteria such as drug-sensitive and drug-resistant *E. Coli* and drug-sensitive *K. pneumonia*. However, except strain B42, none of the other 82 strains showed strong inhibitory activity against Gram-negative strains.

To accelerate the discovery of antibiotics and to early identify the potential antibacterial mechanism of 83 selected strains, a double fluorescent protein reporter system (pDualrep2) [48] was implemented. The *E. coil* cell transformed by the plasmid pDualrep2 is a sensor for translation inhibitors, which can be monitored by induction of Katushka2S expression, and DNA damaging agents, monitored by induction of RFP expression. A typical translation inhibitor erythromycin could induce Katushka2S expression, while levofloxacin, an inhibitor of topoisomerase, elicits expression of RFP. In this study, Katushka2S expression could be induced by S*treptomyces* strain B441 and *Micromonospora* strain B704. RFP expression could be induced by four *Streptomyces* strains, including strain B475, B486, B353, and B98.

Comprehensive analysis of two groups of bioassay data, four *Streptomyces* strains (B475, B486, B353, and B98) showed positive results in antibacterial and pDualrep2 assay simultaneously. It indicated that they are the potential candidates for new antibiotics and deserved to be further investigated for their bioactive secondary metabolic profiles. Out of four *Streptomyces* strains, B475 was selected as an example to explore its secondary metabolites firstly.

Dereplication is crucial to the fast discovery of novel natural products in the extracts of microbial fermentation broths [49]. A large number of secondary metabolites of *Streptomyces* spp. are already known [7]. Therefore, it is necessary to put efforts in rapid and early dereplication on the crude extract of *Streptomyces* strain B475. After analyzing by UPLC-UV-HRESIMS/MS against Chemspider database and comparing the characteristic UV spectra with data in literature, the main bioactive component of *Streptomyces* B475 was identified as echinomycin. The result was in accordance with the screening result of crude extract of B475 on the double fluorescent protein reporter system, since echinomycin is widely known as the first DNA bisintercalator [50,51] and definitely can activate an SOS-response to induce expression of RFP in pDualrep2.

Echinomycin is a cyclic octapeptide that belongs to the family of quinoxaline, a group of antibiotics containing the quinoxaline ring [39,51]. Echinomycin exhibited many interesting bioactivities [52,53,54], but failed in Phase II trials for cancer treatment due to its minimal to no activity in vivo against several tumors [55]. Recently, researches refocused on its significant activity against Gram-positive bacteria. The in vitro bactericidal assay showed echinomycin exhibited strong activity against several clinical isolates of vancomycin-resistant *Enterococci* (VRE), suggesting that it might be useful as anti-VRE drugs [56]. Studies on its in vitro and in vivo antistaphylococcal activities indicated the activity of echinomycin against *S. aureus* including MRSA, was at least equivalent to that of vancomycin [57]. In addition, another study showed that echinomycin had strong inhibitory activities in vitro against drug-resistant and biofilm-forming *S. aureus* and *Enterococcus faecalis* (*E. faecalis*) [58]. All those antibacterial properties against Gram-positive bacteria attracted many researchers to synthesize new analogs of echinomycin [59,60] and discover new members of quinoxaline. To the best of our knowledge, at least 37 members of quinoxaline family have been isolated from a variety of actinobacterial strains (Table S5).

To discover more new analogs of echinomycin in the cultural broth of strain B475, a web platform, Global Natural Products Social Molecular Networking (GNPS; http://gnps.ucsd.edu), was involved [22]. Several studies have reported the successful application of it for effective chemical dereplication and novel metabolite discovery [20,21,61,62,63]. In this study, two clusters (cluster A and B) related to quinoxaline antibiotics were found in the extract of *Streptomyces* B475. It was probably the presence of two distinct chemical modifications that resulted in more than one difference in the MS/MS fragmentation patterns between clusters A and B, suggesting compounds of clusters A and B probably belong to different subgroups. The limitation of molecular networking was unable to align spectra from molecules that differ by two distinct structural modifications according to the GNPS web documentation [64]. Besides, the further purification and structural elucidation of five new putative analogues of quinoxalines are still under way.

## 4. Materials and Methods

### 4.1. Collection of Mangrove Soil Sample

**Figure 9:**
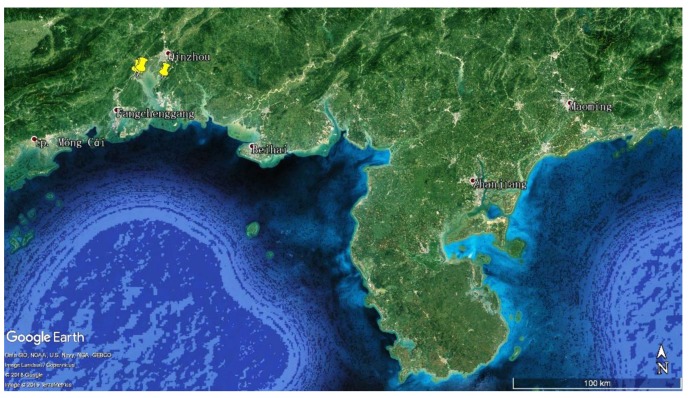
Geographic distribution of sampling sites (yellow) in the Maowei Sea Mangrove Reserve, Qinzhou City, Guangxi Zhuang Autonomous Region, China.

Eight mangrove soil samples were collected from the Maowei Sea Mangrove Reserve, Qinzhou City, Guangxi Zhuang Autonomous Region, China in July 2017 (Figure 9). The information for the samples is listed in Table S6. All the samples were packed in sterilized envelopes and brought to the laboratory at the earliest possible time. Each sample was immediately air-dried in the laminar flow hood before grinding with mortar and pestle.

### 4.2. Isolation and Maintenance of Actinobacteria

Eight culture media were prepared to isolate the actinobacterial strains (Table S1). Culture media were supplemented with nalidixic acid (20 mg/L), cycloheximide (50 mg/L), and potassium dichromate (50 mg/L) to inhibit the growth of Gram-negative bacteria and fungi.

Actinobacteria were isolated by using the dilution plating technique, as described by Li et al. [38]. A volume of 0.2 mL of 10^−2^ (g/mL) soil suspension was spread onto isolation agar plates. After incubation at 28 °C for 2–4 weeks, a single colony was picked up and streaked on the freshly prepared ISP 2 medium (Difco, Becton, Dickinson and Company, Sparks, MD, USA) to obtain the pure isolates. The pure cultures were maintained on ISP 2 agar slants at 4 °C and also preserved in glycerol suspensions (20% v/v) at −80 °C.

### 4.3. PCR Amplification and Sequencing of 16S rRNA Gene

Genomic DNA was extracted from pure isolates as described by Zhou et al. [65] and used as a template to amplify the 16S rRNA gene by PCR with the universal primers 27F (5ʹ-AGAGTTTGATCMTGGCTCAG-3ʹ) and 1492R (5ʹ-GGTTACCTTGTTACGACTT-3ʹ). The reaction mixture (50 μL) contained 25 μL 2×supermix (TransGen Biotech, Beijing, China), 1 μL each of the primers (10 mM, Sangon Biotech, Shanghai, China), 1.5 μL DNA, and 21.5 μL ddH_2_O. The PCR amplification included the following parameters: (i) 95 °C for 3 min (initial denaturation), (ii) 30 cycles of 94 °C for 1 min (denaturation), 60 °C for 1 min (annealing), 72 °C for 1 min (extension), and (iii) 72 °C for 10 min (final extension). The amplicons were then visualized by gel electrophoresis using 5 μL of PCR product in a 1% agarose gel. The PCR products were purified and then sequenced on the ABI PRISM^TM^ 3730XL DNA Analyzer (Thermo Fisher Scientific, Waltham, MA, USA).

### 4.4. Sequence Analysis

The 16S rRNA gene sequences obtained were compared with those from the type strains available in GenBank NCBI (http://www.ncbi.nlm.nih.gov/) and the EzBioCloud database (https://www.ezbiocloud.net/) [66] using the Basic Local Alignment Search Tool (BLAST) [67] for searching the closest match sequence. The corresponding sequences of closely related type species were retrieved from the GenBank database using the EzBioCloud server. Multiple alignments were made using the Clustal_X tool in MEGA version 7.0 [68,69]. A phylogenetic tree based on the neighbor-joining method [70] was constructed under the Kimura’s two-parameter model [71], and bootstrap analyses with 1000 replications [72] were performed with the MEGA version 7.0.

### 4.5. Nucleotide Sequence Accession Numbers

The 16S rRNA sequences of isolated strains were deposited in GenBank under the accession numbers: MN199467-MN199548 and MN204487.

### 4.6. Antibacterial Activity Screening

Based on the analysis of phenotypic and phylogenetic characteristics, only 83 strains were selected to examine their antibacterial potentials. Each strain was transferred to 500 mL Erlenmeyer flasks containing 100 mL of ISP 2 medium and cultivated for 7 days at 28 °C with shaking at 180 rpm. The 300 mL (3 × 100 mL) fermentation broth obtained from each of the isolates was centrifuged at 4500 rpm for 20 min to separate the mycelium portion. The supernatants were extracted twice with ethyl acetate (1:1, v/v). The organic layer was dried up by rotary evaporation, and the residue was dissolved in 3 mL of methanol. The mycelium portion was soaked in acetone for overnight and then filtered. The filtrate was concentrated under vacuum and dissolved in 3 mL of 50 % methanol-water (1:1, v/v). Finally, each strain yielded two kinds of samples for antibacterial assay by the disc diffusion method. The methanol sample (60 µL) was dripped on paper disk (diameter, 6 mm). Besides, 60 µL methanol was used as the negative control, and levofloxacin solution (10 µL, 0.1 mg/mL) was used as the positive control. After being dried in the biosafety hood, the paper disks were transferred to agar plates containing pathogenic bacteria and incubated at 37 °C for 24 h. Finally, the antibacterial activity was evaluated by measuring the diameters of the inhibition zone with Vernier caliper. The indicator bacteria used for antimicrobial assay were six sets of “ESKAPE” bacteria including *Enterococcus faecalis* (ATCC 33186 and 310682), *Staphylococcus aureus* (ATCC 29213 and ATCC 33591), *Klebsiella pneumonia* (ATCC 10031 and ATCC 700603), *Acinetobacter baumannii* (2799 and ATCC 19606), *Pseudomonas aeruginosa* (ATCC 27853 and 2774) and *Escherichia coli* (ATCC 25922 and ATCC 35218), each set consisted of two strains, one was sensitive strain (the former) and the other was drug-resistant strain (the latter). Isolate 310682 was resistant to vancomycin; meanwhile, isolate 2774 was resistant to aminoglycosides and carbapenems. Indicator bacteria were obtained from American Type Culture Collection (ATCC) or the clinic and deposited in the Institute of Medicinal Biotechnology, Chinese Academy of Medical Sciences and Peking Union Medical College.

### 4.7. Mechanism of Action Determination

Ribosome and DNA biosynthesis inhibitors were screened by the double fluorescent protein reporter system with reporter strain JW5503-pDualrep2 [48]. In Brief, 100 µL of ethyl acetate extract was dried up, and 100 µL DMSO was added as the testing sample. A volume of 2 µL of the sample was applied to agar plate containing a lawn of the reporter strain. After overnight incubation at 37 °C, the plate was scanned by ChemiDoc Imaging System (Bio-Rad Laboratories, USA) with two channels, “Cy3-blot” (553/574 nm, green pseudocolor) for RFP fluorescence and “Cy5-blot” (588/633 nm, red pseudocolor) for Katushka2S fluorescence. Induction of expression of Katushka2S is triggered by translation inhibitors, while RFP is upregulated by induction of DNA damage SOS response. Levofloxacin and erythromycin were used as positive controls for DNA biosynthesis and ribosome inhibitors, respectively.

### 4.8. Identification of Bioactive Bands by Thin Layer Chromatography (TLC)

After centrifugation at 3000 rpm for 5 min, 200 µL of crude EA extract of strain B475 was loaded as 80 mm band length on the 20 cm × 10 cm precoated silica gel 60 F_254_ TLC plate (Merck KGaA, Darmstadt, Germany) under a stream of nitrogen using a CAMAG Linomat 5 semi-automatic sample applicator fitted with a 500 µL Hamilton syringe. The plate was developed up to 190 mm with 20 mL Dichloromethane: Methanol = 10:1 (v/v) as mobile phase in a 20 cm × 20 cm CAMAG twin trough chamber previously saturated for 15 min. After development, the plate was removed from the chamber and dried in air. The images of the separated bands were captured and marked by a pencil at UV 254 nm under the CAMAG TLC Visualizer 2. Finally, one-third of the silica gel of each band was scraped respectively, and then cumulated on the agar plates containing methicillin-resistant *Staphylococcus aureus* ATCC 33591 in the biosafety cabinet. The plates were incubated at 37 °C for 24 h, the diameter of the inhibition zones of bands was measured by Vernier caliper.

### 4.9. Identification of Bioactive Peaks by High Performance Liquid Chromatography (HPLC)

The remaining silica gel of the bioactive band was scraped from the TLC plate and then eluted with 20 mL acetone. The acetone eluate was dried under vacuum, and the residues were dissolved in 0.5 mL HPLC grade methanol. After centrifuged at 12,000 rpm for 5 min, 200 µL of the methanol solution was subjected to HPLC (Shimadzu LC-20AT) equipped with Agilent ZORBAX SB-C18 column (9.4 × 250 mm, 5 µm). The mobile phase was acetonitrile-water solution at the flow rate of 2 ml/min in gradient elution method: 10% acetonitrile for 0–5 min; 10% to 70% acetonitrile for 5–45 min; 70% to 90% acetonitrile for 45–55 min; 90% acetonitrile for 55–60 min. The detection wavelength used was 215 nm. For identification of the bioactive peaks, the fraction collected per minute and yielded 58 fractions (0–3 min as fraction 1). All fractions were dried under vacuum and dissolved in 50 μL methanol. Twenty microliters of each fraction was screened against methicillin-resistant *Staphylococcus aureus* ATCC 33591 by the disc diffusion method. After incubated at 37 °C for 24 h, the diameters of the inhibition zones of bands were measured by Vernier caliper.

### 4.10. Dereplication and Molecular Network Analysis of Microbial Extracts

Three microliters of samples from strain B475, including the EA crude extract, the active band from TLC, and the active peaks from HPLC, were analyzed by UPLC-HRESIMS/MS (Waters Xevo G2-XS QTof) equipped with ACQUITY UPLC BEH C18 column (2.1 × 100 mm, 1.7 μm). The column was eluted with a gradient mobile phase of acetonitrile-water solution at the flow rate of 0.3 mL/min: 10% acetonitrile for 2 min, 10% to 70% acetonitrile for following 28 min, then 70% to 90% acetonitrile for 5 min, finally, 90% acetonitrile for 3 min. The PDA detector was set between 194 and 400 nm. Source operating parameters were defined as below: capillary and cone voltages were set at 2.0 KV and 40 V, desolvation temperature and the source temperature were set at 260 °C and 100 °C, respectively. High-purity nitrogen was used as the nebulizer and auxiliary gas. Cone gas was set to a flow rate of 50 L/h, and the desolvation gas flow was maintained at 600 L/h. Argon was used as the collision gas. Mass accuracy was maintained by using a lock spray with leucine-enkephalin (in positive ion mode [M+H]^+^ = 556.2771 Da) at a concentration of 200 pg/mL and a flow rate of 10 μL/min as reference.

MS methods were acquired by data-independent acquisition mode (MS^E^) for UNIFI analysis and data-dependent acquisition (DDA) for GNPS analysis, respectively. MS^E^ was carried out by operating the instrument at positive ion mode, applying the MS and MS/MS functions with 6 V low energy and 20–45 V high energy collision to collect the mass to charge ratio (m/z) from 100 to 1500 Da. MassLynx V4.1 software was used in data acquisition, and UNIFI software was used for processing data and dereplication by searching the predicted accurate mass against the Chemspider (http://www.chemspider.com/). DDA was performed in positive ion mode, the full MS survey scan was performed for 0.1 s time in the range of 100–1600 Da, while MS/MS scanned over a mass range of 50–1600 Da by the same scan time. The eight most intense ions were further scanned for MS/MS fragmentation spectra. The gradient of collision energy was set as 6 to 9 V for LM CE (low-mass collision energy) and 80 to 90 V for HM CE (high-mass collision energy). Automatic switching to MS/MS mode was enabled when TIC intensity rose above 10,000 counts and switching off when 0.2 s had elapsed, or TIC intensity was 1000 counts. Tolerance window of ±3.0 Da was set in deisotope peak detection mode. Dynamic Peak Exclusion was enabled, acquired, and then excluded for 0.2 s. Raw data files obtained from DDA acquisition were converted to the 32-bit mzML format with MS-Convert [73] and then uploaded on the GNPS web platform for dereplication and molecular networking construction. The MS/MS molecular network was generated using the GNPS “Classic” molecular networking workflow (METABOLOMICS-SNETS-V2), in which the MS-Cluster was activated. Parameters for molecular network generation were set as follows: precursor mass tolerance m/z 0.02 Da, MS/MS fragment ion tolerance m/z 0.04 Da, minimum cosine score 0.65, minimum matched fragment ions 4, minimum cluster size 2, network TopK 10; the spectral library matching was performed with the same minimum cosine score and six matched fragment ion number filter parameters. The generated molecular network was visualized using Cytoscape 3.7.1 [74] and searched for clusters of *m/z* data that clarify the compounds generating the clusters.

### 4.11. Accumulation and Measurement of Peak 3

The remaining EA extract of strain B475 was subjected to TLC, as showed above (Materials and Methods 4.8), and further purified by HPLC, as mentioned above (Materials and Methods 4.9). Finally, the bioactive fractions at R_t_ = 45–46 min were pooled and concentrated by rotary evaporation under vacuum to yield the peak **3** (0.7 mg). Peak **3** was dissolved in CDCl_3_ and measured by a Varian VNS-600 NMR instrument at 600 MHz with TMS as the internal standard to obtain the ^1^H NMR spectrum.

## 5. Conclusions

In this study, we demonstrated that under-explored mangrove soil in the Maowei Sea Mangrove Reserve of China is a valuable source for the isolation of actinobacteria, in terms of abundance, and diversity. Our study suggests that mangrove-derived actinobacteria have potential to produce promising new bioactive secondary metabolites which can be explored efficiently through the combination of multiple discovery strategies. Actinobacteria originated from Maowei Sea Mangrove Reserve is just an example. Vast mangrove resources distributed in different regions all over the world are untapped and can be proved as treasure for novel actinobacterial strains. However, the question remains how to find those new antibiotics. We hope that our discovery strategies might open a new path to capture new antibiotics in the mangrove.

## Figures and Tables

**Table 1:** Information on genera distribution of actinobacterial strains in this study.

Genus	Total Number of Actinobacterial Strains				
	Isolation	Assay	Positive in Assay	RFP	Katushka2S
*Micromonospora*	126	35	14	0	1
*Streptomyces*	38	19	10	4	1
*Agromyces*	24	5	3	0	0
*Microbispora*	15	1	0	0	0
*Actinomadura*	13	4	0	0	0
*Rhodococcus*	11	3	0	0	0
*Nocardia*	9	3	1	0	0
*Mycobacterium*	6	1	0	0	0
*Kitasatospora*	4	1	1	0	0
*Paenarthrobacter*	4	2	0	0	0
*Sinomonas*	3	1	0	0	0
*Pseudarthrobacter*	1	1	0	0	0
*Micrococcus*	1	1	0	0	0
*Intrasporangium*	1	1	1	0	0
*Gordonia*	1	1	0	0	0
*Nakamurella*	1	1	1	0	0
*Actinocorallia*	1	1	0	0	0
*Arthrobacter*	1	1	0	0	0
*Mycolicibacterium*	1	1	1	0	0
Total number	261	83	32	4	2

## Supplementary Materials

The following are available online at https://www.mdpi.com/2079-6382/8/4/236/s1, Table S1: Compositions of eight media used for isolation of mangrove actinobacteria; Table S2: Information on genera distribution of actinobacterial isolates from different samples; Table S3: Information on genera distribution of actinobacterial isolates recovered from the different culture media; Table S4: Antimicrobial activities of cultivable actinobacteria from Maowei Sea mangrove soil and their similarity of 16S rRNA gene sequences (>750 bp); Table S5: The Quinoxaline-type antibiotics isolated from actinobacteria; Table S6: Information of soil samples; Figure S1: ^1^H NMR (600 MHz) spectrum of peak **3** in CDCl_3_; Figure S2: The UPLC-UV-HRESIMS/MS analysis and dereplication of peak **2**.
