# Supplementary material for: Exploitation of Potentially New Antibiotics from Mangrove Actinobacteria in Maowei Sea by Combination of Multiple Discovery Strategies
Source: Antibiotics (Basel). 2019 Nov 27;8(4):236. doi: 10.3390/antibiotics8040236 (PMC6963217; doi:10.3390/antibiotics8040236)
Supplement: Supplementary file 1 [file antibiotics-08-00236-s001.pdf]

antibiotics

MDPI

Supplementary Materials.

Exploitation of Potentially New Antibiotics from Mangrove Actinobacteria in Maowei Sea by Combination of Multiple Discovery Strategies

Qin-Pei Lu [1,2,†], Jing-Jing Ye [3,†], Yong-Mei Huang [4], Di Liu [5], Li-Fang Liu [1,2], Kun Dong [6], Elizaveta A. Razumova [7], Ilya A. Osterman [7,8], Petr V. Sergiev [7,8], Olga A. Dontsova [7,8,9], Shu-Han Jia [3], Da-Lin Huang [3,*] and Cheng-Hang Sun [1,2,*]

[1] Department of Microbial Chemistry, Institute of Medicinal Biotechnology, Chinese Academy of Medical Sciences & Peking Union Medical College, Beijing 100050, China
[2] Beijing Key Laboratory of Antimicrobial Agents, Institute of Medicinal Biotechnology, Chinese Academy of Medical Sciences & Peking Union Medical College, Beijing 100050, China
[3] College of Basic Medical Sciences, Guilin Medical University, Guilin 541004, China
[4] Zhanjiang for R&D Marine Microbial Resources in the Beibu Gulf Rim, Marine Biomedical Research Institute, Guangdong Medical University, Zhanjiang 524023, China
[5] College of Life Sciences, Jiamusi University, Jiamusi 154007, China
[6] College of Life Science and Technology, China Pharmaceutical University, Nanjing 211198, China
[7] Department of Chemistry, Lomonosov Moscow State University, Moscow 119992, Russia
[8] Center of Life Sciences, Skolkovo Institute of Science and Technology, Moscow 143025, Russia
[9] Shemyakin-Ovchinnikov Institute of Bioorganic Chemistry, Russian Academy of Sciences, Moscow 119992, Russia
* Correspondence: chenghangsun@hotmail.com, sunchenghang@imb.pumc.edu.cn (C.-H.S.); huangdalin@glmc.edu.cn (D.-L.H.); Tel.: +86-10-63165272 (C.-H.S.)
† These authors contributed equally to this work.

List of Tables and Figures

Table S1. Compositions of eight media used for isolation of mangrove actinobacteria.
Table S2. Information on genera distribution of actinobacterial isolates from different samples.
Table S3. Information on genera distribution of actinobacterial isolates recovered from the different culture media.
Table S4. Antimicrobial activities of cultivable actinobacteria from Maowei Sea mangrove soil and their similarity of 16S rRNA gene sequences (>750 bp).
Table S5. The Quinoxaline-type antibiotics isolated from the actinobacteria.
Table S6. Information of soil samples.
Figure S1. ^{1}H NMR (600 MHz) spectrum of peak **3** in $CDCl_3$.
Figure S2. The UPLC-UV-HRESIMS/MS analysis and dereplication of peak **2**.

Table 1. Compositions of eight media used for isolation of mangrove actinobacteria.

No.	Name	Composition
M1	modified ISP 2 agar medium	Glucose (4.00 g); yeast extract powder (4.00 g); malt extract powder (5.00 g); trace salt mixture (1.00 ml); vitamin mixture (1.00 ml); agar (15.00 g); distilled water (1000.00 ml); pH= 7.2
M2	Cochrane 2 medium	Glucose (10.00 g); peptone (5.00 g); tryptone (3.00 g); NaCl (5.00 g); vitamin mixture (1.00 ml); agar (15.00 g); distilled water (1000.00 ml); pH= 7.2
M3	GA medium	Soluble starch (20.00 g); KNO_3 (1.00 g); K_2HPO_4 (0.50 g); $MgSO_4·7H_2O$ (0.50 g); $FeSO_4·7H_2O$ (0.01 g); Salt (3.00 g); agar (15.00 g); distilled water (1000.00 ml); pH= 7.2
M4	NA medium	Peptone (10.00 g); NaCl (5.00 g); beef extract (3.00 g); agar (15.00 g); distilled water (1000.00 ml); pH= 7.2
M5	Czapek'medium	HNO_3 (3.00 g); K_2HPO_4 (1.00 g); $MgSO_4$ (0.50 g); KCl (0.50 g); $FeSO_4$ (0.01 g); sucrose (30.00 g); sea salt crystal (3.00 g); agar (15.00 g); distilled water (1000.00 ml); pH= 7.2
M6	Trehalose-proline medium	Trehalose (5.00 g); proline (1.00 g); $(NH4)_2SO_4$ (1.00 g); NaCl (1.00 g); $CaCl_2$ (2.00 g); K_2HPO_4 (1.00 g); $MgSO_4$ (1.00 g); vitamin mixture (1.00 ml); agar (15.00 g); distilled water (1000.00 ml); pH= 7.2
M7	Raffinose-Histidine medium	Raffinose (5.00 g); L-histidine (1.00 g); KNO_3 (1.00 g); NaCl (1.00 g); $CaCl_2$ (2.00 g); K_2HPO_4 (1.00 g); $MgSO_4$ (1.00 g); agar (15.00 g); distilled water (1000.00 ml); pH= 7.2
M8	R_2A medium	R_2A; seawater (300.00 ml); distilled water (700.00 ml); agar(15.00 g); pH= 7.2

Trace salt mixture: $FeSO_4·7H_2O$ (0.20 g); $MnCl_2·4H_2O$ (0.01 g); $ZnSO_4·7H_2O$ (0.01 g); distilled water (100.00 mL). vitamin mixture: thiamine (0.10 g); pyridoxine (0.10 g); riboflavin (0.10 g); niacin (0.10 g); biotin (0.10 g); distilled water (100.00 mL).

Table 2. Information on genera distribution of actinobacterial isolates from different samples.

Genera	Samples							
	S1	S2	S3	S4	S5	S6	S7	S8
Streptomyces	3	1	1	3	13	3	12	2
Kitasatospora	1	1	-	-	1	-	1	-
Agromyces	-	2	1	-	11	-	5	5
Sinomonas	-	-	-	1	1	1	-	-
Micrococcus	-	-	-	-	1		-	-
Pseudarthrobacter	-	-	-	1	-	-	-	-
Paenarthrobacter	-	-	1	-	2	-	1	-

Arthrobacter	-	-	-	1	-	-	-	-
Intrasporangium	-	-	-	1	-	-	-	-
Micromonospora	15	12	8	25	42	12	3	9
Rhodococcus	-	-	-	3	4	-	3	1
Nocardia	-	-	-	1	2	-	5	1
Gordonia	-	-	-	-	1	-	-	-
Mycobacterium	-	-	-	1	3	-	1	1
Mycolicibacterium	-	-	-	-	-	-	1	-
Microbispora	2	1	6	3	1	-	1	1
Actinomadura	-	-	2	8	1	-	1	1
Actinocorallia	-	-	-	-	-	-	-	1
Nakamurella	-	-	1	-	-	-	-	-
Total number of genera	4	5	7	11	13	3	11	9
Total number of isolates	21	17	20	48	83	16	34	22

Note: −, no isolate.

Table 3. Information on genera distribution of actinobacterial isolates recovered from the different culture media.

**Genera**	**Media**							
	**M1**	**M2**	**M3**	**M4**	**M5**	**M6**	**M7**	**M8**
Streptomyces	1	4	11	1	4	5	7	5
Kitasatospora	-	–	2	-	1	1	-	-
Agromyces	4	2	4	2	-	-	7	5
Sinomonas	-	-	3	-	-	-	-	-
Micrococcus	-	-	-	-	-	1	-	-
Pseudarthrobacter	-	-	-	-	1	-	-	-
Paenarthrobacter	1	-	1	-	-	-	1	1
Arthrobacter	-	-	-	-	-	-	-	1
Intrasporangium	-	1	-	-	-	-	-	-
Micromonospora	12	19	50	-	10	7	21	7
Rhodococcus	-	-	3	-	1	2	2	3
Nocardia	-	-	2	1	-	2	1	3
Gordonia	-	-	-	-	-	-	1	-
Mycobacterium	-	-	4	-	-	1	-	1
Mycolicibacterium	-	-	1	-	-	-	-	-

Microbispora	-	-	11	-	2	2	-	-
Actinomadura	-	1	5	-	-	6	1	-
Actinocorallia	-	-	-	-	-	1	-	-
Nakamurella	-	-	-	-	1	-	-	-
Total number of genera	4	5	12	3	7	10	8	8
Total number of isolates	18	27	97	4	20	28	41	26

Note: −, no isolate.

Table 4. Antimicrobial activities of cultivable actinobacteria from Maowei Sea mangrove soil and their similarity of 16S rRNA gene sequences (>750 bp).

Isolate [a] (GenBank Accession No.)	Genus and Species (Similarity, %)	Samples [b]	Activity [c]											
			E. coli		*P. eruginosa*		*K. pneumonia*		*A. baumannii*		*E. faecalis*		*S. aureus*	
			S	R	S	R	S	R	S	R	S	R	S	R
B486(MN199470)$^{+-}$	*Streptomyces seoulensis* NRRL B-24310^{T} (99.93%)	E	-	-	-	-	-	-	1.10	0.80	1.50	1.54	1.90	2.70
		M	1.20	-	-	-	-	-	1.10	-	1.60	1.76	2.10	2.55
B121(MN199471)	*Micromonospora mirobrigensis* DSM 44830^{T} (99.47%)	E	-	-	-	-	-	-	-	-	-	-	-	-
		M	-	-	-	-	-	--	-	-	-	-	-	-
B65(MN199518)$^{+}$	*Micromonospora harpali* NEAU-JC6^{T} (100%)	E	-	-	-	-	-	-	-	-	-	1.20	-	-
		M	-	-	-	-	-	-	-	-	-	-	-	-
**B290(MN199529)**$^{+}$	*Streptomyces viridobrunneus* LMG 20317^{T} (100%)	E	-	-	-	-	-	-	-	-	-	1.50	2.10	2.50
		M	-	-	-	-	-	-	-	-	-	1.20	1.40	1.85
**B448(MN199472)** $^{+-}$	*Streptomyces albaduncus* JCM 4715^{T} (100%)	E	1.00	-	-	-	1.05	-	1.00	-	-	1.30	2.10	-
		M	-	-	-	-	1.25	-	-	-	-	0.90	1.95	-
**B42(MN199473)** $^{+-}$	*Micromonospora tulbaghiae* DSM 45142^{T} (100%)	E	2.35	2.25	-	-	2.60	-	1.45	-	-	-	3.00	-
		M	-	-	-	-	-	-	-	-	-	-	1.30	1.50
**B475(MN199475)** $^{+-}$	*Streptomyces seoulensis* NRRL B-24310^{T} (99.93%)	E	-	-	-	-	-	-	1.45	-	1.75	2.15	2.35	3.60
		M	-	1.10	-	-	-	-	1.25	-	1.95	2.65	2.40	2.60
B415(MN199474)	*Micromonospora maritima* D10-9-5^{T} (99.87%)	E	-	-	-	-	-	-	-	-	-	-	-	-
		M	-	-	-	-	-	-	-	-	-	-	-	-
B401(MN199476)	*Micromonospora oryzae* CP2R9-1^{T} (99.20%)	E	-	-	-	-	-	-	-	-	-	-	-	-
		M	-	-	-	-	-	-	-	-	-	-	-	-
**B331(MN199477)** $^{+}$		E	-	-	-	-	-	-	-	-	-	-	1.40	2.60

	Micromonospora schwarzwaldensis HKI0641^{T} (99.87%)	M	-	-	-	-	-	-	-	-	-	-	-	1.40
B704(MN199478)$^{+-}$	*Micromonospora auratinigra* DSM 44815^{T} (98.97%)	E	-	-	-	-	1.10	-	1.30	-	1.70	1.80	1.40	-
		M	-	-	-	-	-	-	-	-	-	-	1.10	-
B452(MN199521)	*Micromonospora wenchangensis* CCTCC AA 2012002^{T} (99.07%)	E	-	-	-	-	-	-	-	-	-	-	-	-
		M	-	-	-	-	-	-	-	-	-	-	-	-
B285(MN199479)$^{+}$	*Micromonospora mirobrigensis* DSM 44830 ^{T}(100%)	E	-	-	-	-	-	-	-	-	-	-	-	-
		M	-	-	-	-	-	-	-	-	-	1.30	-	-
B490(MN199480)	*Streptomyces wuyuanensis* CGMCC 4.7042^{T} (100%)	E	-	-	-	-	-	-	-	-	-	-	-	-
		M	-	-	-	-	-	-	-	-	-	-	-	-
B122(MN199468)$^{+}$	*Micromonospora saelicesensis* Lupac 09^{T} (99.19%)	E	-	-	-	-	-	-	-	-	-	-	-	1.30
		M	-	-	-	-	-	-	-	-	-	-	-	1.20
B649(MN199481)$^{-}$	*Mycolicibacterium poriferae* ATCC 35087^{T} (98.93%)	E	-	-	-	-	-	-	-	0.70	-	-	-	-
		M	-	-	-	-	-	-	-	-	-	-	-	-
B593(MN199482)$^{-}$	*Agromyces binzhouensis* OAct353^{T} (98.29%)	E	-	-	-	-	-	-	-	0.65	-	-	-	-
		M	-	-	-	-	-	-	-	-	-	-	-	-
B300(MN199483)$^{-}$	*Agromyces binzhouensis* OAct353^{T} (98.36%)	E	-	-	-	-	-	-	-	0.75	-	-	-	-
		M	-	-	-	-	-	-	-	-	-	-	-	-
B323(MN199484)	*Micromonospora chalcea* DSM 43026^{T} (99.73%)	E	-	-	-	-	-	-	-	-	-	-	-	-
		M	-	-	-	-	-	-	-	-	-	-	-	-
B74(MN199485)$^{+}$	*Micromonospora aurantiaca* ATCC 27029^{T} (99.87%)	E	-	-	-	-	-	-	-	-	0.65	1.30	1.10	-
		M	-	-	-	-	-	-	-	-	-	0.80	-	-
B276(MN199486)$^{+-}$	*Agromyces binzhouensis* OAct353^{T} (98.37%)	E	-	-	-	-	-	-	-	0.65	-	-	-	-
		M	-	-	-	-	-	-	-	-	-	1.10	0.75	-
B31(MN199487)	*Agromyces binzhouensis* OAct353^{T} (98.40%)	E	-	-	-	-	-	-	-	-	-	-	-	-
		M	-	-	-	-	-	-	-	-	-	-	-	-
B610(MN199488)	*Actinomadura Mexicana* A290^{T} (99.87%)	E	-	-	-	-	-	-	-	-	-	-	-	-
		M	-	-	-	-	-	-	-	-	-	-	-	-
B211(MN204487)$^{+}$	*Intrasporangium flavum* MUSC 78^{T} (98.93%)	E	-	-	-	-	-	-	-	-	1.00	-	-	-
		M	-	-	-	-	-	-	-	-	-	-	-	-

B581(MN199489) +	*Kitasatospora aureofaciens* ATCC 10762ᵀ (100%)	E	-	-	-	-	-	-	-	-	-	-	-	-
		M	-	-	-	-	-	-	-	-	-	-	0.85	-
**B390(MN199540)** +-	*Streptomyces viridobrunneus* LMG 20317ᵀ (100%)	E	-	-	-	-	-	-	1.10	-	1.50	-	1.90	2.70
		M	1.20	-	-	-	-	-	1.10	-	1.60	-	2.10	2.55
**B409(MN199526)** +-	*Micromonospora schwarzwaldensis* HKI0641ᵀ (99.87%)	E	-	-	-	-	1.30	-	1.40	-	1.20	1.45	1.80	2.25
		M	-	-	-	-	-	-	-	-	-	-	1.00	1.35
B346(MN199490)	*Paenarthrobacter ureafaciens* DSM 20126ᵀ (99.87%)	E	-	-	-	-	-	-	-	-	-	-	-	-
		M	-	-	-	-	-	-	-	-	-	-	-	-
B94(MN199532)	*Micromonospora maritima* D10-9-5ᵀ (99.73%)	E	-	-	-	-	-	-	-	-	-	-	-	-
		M	-	-	-	-	-	-	-	-	-	-	-	-
B309(MN199545)	*Streptomyces youssoufiensis* X4ᵀ (100%)	E	-	-	-	-	-	-	-	-	-	-	-	-
		M	-	-	-	-	-	-	-	-	-	-	-	-
B598(MN199491)	*Micromonospora soli* SL3-70ᵀ (99.73%)	E	-	-	-	-	-	-	-	-	-	-	-	-
		M	-	-	-	-	-	-	-	-	-	-	-	-
B731(MN199492)	*Nocardia nova* NBRC 15556ᵀ (100%)	E	-	-	-	-	-	-	-	-	-	-	-	-
		M	-	-	-	-	-	-	-	-	-	-	-	-
B419(MN199493) -	*Streptomyces caeni* HA15995ᵀ (99.73%)	E	-	-	-	-	-	-	-	-	-	-	-	-
		M	-	-	1.80	-	-	-	-	-	-	-	-	-
**B353(MN199494)** +-	*Streptomyces seoulensis* NRRL B-24310ᵀ (99.93%)	E	-	-	-	-	-	-	1.40	-	1.65	1.80	3.20	3.50
		M	-	-	-	-	-	-	1.35	0.75	1.80	2.00	2.60	3.80
**B277(MN199495)** +	*Micromonospora terminaliae* TMS7ᵀ (99.73%)	E	-	-	-	-	-	-	-	-	-	1.15	1.70	2.40
		M	-	-	-	-	-	-	-	-	-	-	-	1.35
B101(MN199496) +	*Micromonospora yasonensis* DS3186ᵀ (99.33%)	E	-	-	-	-	-	-	-	-	-	-	-	-
		M	-	-	-	-	-	-	-	-	-	1.90	-	-
**B98(MN199497)** +-	*Streptomyces albogriseolus* NRRL B-1305ᵀ (100%)	E	-	-	-	-	-	-	1.10	-	1.50	1.90	1.80	1.30
		M	-	-	-	-	-	-	1.20	-	1.60	2.80	2.40	3.20
B434(MN199498)	*Streptomyces malaysiensis* NBRC 16446ᵀ (100%)	E	-	-	-	-	-	-	-	-	-	-	-	-
		M	-	-	-	-	-	-	-	-	-	-	-	-
B451(MN199467)	*Arthrobacter koreensis* CA15-8ᵀ (100%)	E	-	-	-	-	-	-	-	-	-	-	-	-
		M	-	-	-	-	-	-	-	-	-	-	-	-
B432(MN199499)	*Streptomyces tendae* ATCC 19812ᵀ (100%)	E	-	-	-	-	-	-	-	-	-	-	-	-
		M	-	-	-	-	-	-	-	-	-	-	-	-

B459(MN199500)	*Streptomyces misionensis* DSM 40306T (99.47%)	E	-	-	-	-	-	-	-	-	-	-	-	-
		M	-	-	-	-	-	-	-	-	-	-	-	-
B441(MN199501)	*Streptomyces albogriseolus* NRRL B-1305T (100%)	E	-	-	-	-	-	-	-	-	-	-	-	-
		M	-	-	-	-	-	-	-	-	-	-	-	-
B187(MN199522)	*Streptomyces violaceorubidus* LMG 20319T (99.87%)	E	-	-	-	-	-	-	-	-	-	-	-	-
		M	-	-	-	-	-	-	-	-	-	-	-	-
B82(MN199502)	*Micromonospora haikouensis* 232617T (99.87%)	E	-	-	-	-	-	-	-	-	-	-	-	-
		M	-	-	-	-	-	-	-	-	-	-	-	-
B392(MN199503)	*Micromonospora vinacea* GUI63T (99.87%)	E	-	-	-	-	-	-	-	-	-	-	-	-
		M	-	-	-	-	-	-	-	-	-	-	-	-
B81(MN199504)	*Micromonospora oryzae* CP2R9-1T (99.87%)	E	-	-	-	-	-	-	-	-	-	-	-	-
		M	-	-	-	-	-	-	-	-	-	-	-	-
B384(MN199519)	*Micromonospora yasonensis* DS3186T (99.33%)	E	-	-	-	-	-	-	-	-	-	-	-	-
		M	-	-	-	-	-	-	-	-	-	-	-	-
B321(MN199505)	*Microbispora hainanensis* 211020T (99.73%)	E	-	-	-	-	-	-	-	-	-	-	-	-
		M	-	-	-	-	-	-	-	-	-	-	-	-
B573(MN199506)	*Rhodococcus equi* NBRC 101255T (100%)	E	-	-	-	-	-	-	-	-	-	-	-	-
		M	-	-	-	-	-	-	-	-	-	-	-	-
B471(MN199507)	*Rhodococcus zopfii* NBRC 100606T (100%)	E	-	-	-	-	-	-	-	-	-	-	-	-
		M	-	-	-	-	-	-	-	-	-	-	-	-
B502(MN199469)	*Paenarthrobacter ureafaciens* DSM 20126T (99.87%)	E	-	-	-	-	-	-	-	-	-	-	-	-
		M	-	-	-	-	-	-	-	-	-	-	-	-
B310(MN199508)	*Pseudarthrobacter chlorophenolicus* A6T (99.47%)	E	-	-	-	-	-	-	-	-	-	-	-	-
		M	-	-	-	-	-	-	-	-	-	-	-	-
B111(MN199509)	*Gordonia rubripertincta* NBRC 101908T (100%)	E	-	-	-	-	-	-	-	-	-	-	-	-
		M	-	-	-	-	-	-	-	-	-	-	-	-
B463(MN199510) +	*Nakamurella silvestris* S20-107T (100%)	E	-	-	-	-	-	-	-	-	-	-	-	-
		M	-	-	-	-	-	-	-	-	-	0.65	-	-
B651(MN199511)	*Actinocorallia aurantiaca* JCM 8201T (99.47%)	E	-	-	-	-	-	-	-	-	-	-	-	-
		M	-	-	-	-	-	-	-	-	-	-	-	-
B402(MN199535)	*Micromonospora tulbaghiae*	E	-	-	-	-	-	-	-	-	-	-	-	-

	DSM 45142^T (100%)	M	-	-	-	-	-	-	-	-	-	-	-	-
B456(MN199524)	*Micromonospora chalcea* DSM 43026^T (99.87%)	E	-	-	-	-	-	-	-	-	-	-	-	-
		M	-	-	-	-	-	-	-	-	-	-	-	-
B129(MN199520)	*Agromyces indicus* NIO-1018^T (100%)	E	-	-	-	-	-	-	-	-	-	-	-	-
		M	-	-	-	-	-	-	-	-	-	-	-	-
B449(MN199525)$^{+}$	*Streptomyces bungoensis* DSM 41781^T (100%)	E	-	-	-	-	-	-	-	-	-	-	1.70	1.60
		M	-	-	-	-	-	-	-	-	-	-	-	-
B550(MN199534)	*Micromonospora auratinigra* DSM 44815^T (99.33%)	E	-	-	-	-	-	-	-	-	-	-	-	-
		M	-	-	-	-	-	-	-	-	-	-	-	-
B535(MN199527)	*Micromonospora equine* Y22^T (99.33%)	E	-	-	-	-	-	-	-	-	-	-	-	-
		M	-	-	-	-	-	-	-	-	-	-	-	-
B241(MN199523)	*Micromonospora aurantiaca* ATCC 27029^T (100%)	E	-	-	-	-	-	-	-	-	-	-	-	-
		M	-	-	-	-	-	-	-	-	-	-	-	-
B406(MN199516)	*Actinomadura maheshkhaliensis* 13-12-50^T (99.73%)	E	-	-	-	-	-	-	-	-	-	-	-	-
		M	-	-	-	-	-	-	-	-	-	-	-	-
B555(MN199537)	*Actinomadura bangladeshensis* 3-46-b3^T(98.93%)	E	-	-	-	-	-	-	-	-	-	-	-	-
		M	-	-	-	-	-	-	-	-	-	-	-	-
B531(MN199533)	*Actinomadura maheshkhaliensis* 13-12-50^T (99.60%)	E	-	-	-	-	-	-	-	-	-	-	-	-
		M	-	-	-	-	-	-	-	-	-	-	-	-
B14(MN199513)$^{+}$	*Micromonospora mangrovi* 2803GPT1-18^T (99.07%)	E	-	-	-	-	-	-	-	-	-	-	1.05	-
		M	-	-	-	-	-	-	-	-	-	-	-	-
B238(MN199542)$^{+}$	*Micromonospora haikouensis* 232617^T (99.47%)	E	-	-	-	-	-	-	-	-	-	-	-	1.00
		M	-	-	-	-	-	-	-	-	-	-	-	-
B320(MN199530)	*Micromonospora chalcea* DSM 43026^T (99.87%)	E	-	-	-	-	-	-	-	-	-	-	-	-
		M	-	-	-	-	-	-	-	-	-	-	-	-
B136(MN199538)	*Micromonospora profundi* DS3010^T (99.73%)	E	-	-	-	-	-	-	-	-	-	-	-	-
		M	-	-	-	-	-	-	-	-	-	-	-	-
B382(MN199512)	*Micromonospora krabiensis* DSM 45344^T (99.73%)	E	-	-	-	-	-	-	-	-	-	-	-	-
		M	-	-	-	-	-	-	-	-	-	-	-	-
B604(MN199531)	*Micromonospora aurantiaca* ATCC 27029^T (100%)	E	-	-	-	-	-	-	-	-	-	-	-	-
		M	-	-	-	-	-	-	-	-	-	-	-	-
B344(MN199515)	*Sinomonas flava*	E	-	-	-	-	-	-	-	-	-	-	-	-

	CW 108^{T} (99.47%)	M	-	-	-	-	-	-	-	-	-	-	-	-
B623(MN199543) $^{+}$	*Streptomyces misionensis*	E	-	-	-	-	-	-	-	-	-	-	1.10	-
	DSM 40306^{T} (99.47%)	M	-	-	-	-	-	-	-	-	-	-	-	-
B473(MN199544)	*Streptomyces purpureus*	E	-	-	-	-	-	-	-	-	-	-	-	-
	NBRC 13927^{T} (99.47%)	M	-	-	-	-	-	-	-	-	-	-	-	-
B567(MN199528) $^{+}$	*Micromonospora aurantiaca*	E	-	-	-	-	-	-	-	-	1.60	-	-	-
	ATCC 27029^{T} (100%)	M	-	-	-	-	-	-	-	-	-	-	-	-
B413(MN199517) $^{-}$	*Micromonospora krabiensis*	E	-	-	-	-	-	-	1.10	-	-	-	-	-
	DSM 45344^{T} (100%)	M	-	-	-	-	-	-	-	-	-	-	-	-
B12(MN199514)	*Rhodococcus ruber*	E	-	-	-	-	-	-	-	-	-	-	-	-
	DSM 43338^{T} (98.93%)	M	-	-	-	-	-	-	-	-	-	-	-	-
B235(MN199541)	*Micromonospora globbae*	E	-	-	-	-	-	-	-	-	-	-	-	-
	WPS1-2^{T} (99.33%)	M	-	-	-	-	-	-	-	-	-	-	-	-
B158(MN199546)	*Streptomyces pseudogriseolus*	E	-	-	-	-	-	-	-	-	-	-	-	-
	NRRL B-3288^{T} (100.00%)	M	-	-	-	-	-	-	-	-	-	-	-	-
B378(MN199539)	*Nocardia niigatensis*	E	-	-	-	-	-	-	-	-	-	-	-	-
	NBRC 100131^{T} (100%)	M	-	-	-	-	-	-	-	-	-	-	-	-
**B391(MN199536)** $^{+}$	*Nocardia africana*	E	-	-	-	-	-	-	-	-	-	2.05	-	-
	DSM 44491^{T} (99.87%)	M	-	-	-	-	-	-	-	-	-	-	-	-
B644(MN199547)	*Mycobacterium aquiterrae*	E	-	-	-	-	-	-	-	-	-	-	-	-
	S-I-6^{T} (99.33%)	M	-	-	-	-	-	-	-	-	-	-	-	-
B134(MN199548)	*Micrococcus aloeverae*	E	-	-	-	-	-	-	-	-	-	-	-	-
	AE-6^{T} (99.87%)	M	-	-	-	-	-	-	-	-	-	-	-	-
		Methanol	-	-	-	-	-	-	-	-	-	-	-	-
		Levofloxacin	1.17	0.65	1.15	1.00	1.05	0.80	1.60	1.00	2.43	1.20	2.80	2.09

a: $^{+-}$ represents strain against both Gram-Positive and Gram-Negative bacteria; $^{+}$ represents strain against only Gram-Positive bacteria; $^{-}$ represents strain against only Gram-Negative bacteria. Bold number represents strong antibacterial activity (inhibition zone>2.0 cm). b: E represents crude sample extracted with ethyl acetate; M represents crude sample from extracted with acetone from mycelium. c: The diameters of the inhibition zones, cm; the diameters of the paper disk, 0.60 cm; −, no inhibitory activity. Methanol, negative control; Levofloxacin, positive control.

Table 5. The Quinoxaline-type antibiotics isolated from the actinobacteria.

Compound	UVmax (nm)	Molecular Mass	Molecular Formula	Ref.
echinomycin (quinomycin A) [a]	244; 210; 326	1101; $[M+H]^+$	$C_{51}H_{64}N_{12}O_{12}S_2$	[1]
quinomycin B [a]	244; 210; 326	1129; $[M+H]^+$	$C_{53}H_{68}N_{12}O_{12}S_2$	[1]
quinomycin C [a]	244; 210; 326	1157; $[M+H]^+$	$C_{55}H_{72}N_{12}O_{12}S_2$	[1]
quinomycin E [a]	244; 210; 326	1143; $[M+H]^+$	$C_{54}H_{70}N_{12}O_{12}S_2$	[1]
quinomycin I [a]	244; 210; 326	1145; $[M+H]^+$	$C_{53}H_{69}N_{12}O_{13}S_2$	[1]
quinomycin J [a]	244; 210; 326	1173; $[M+H]^+$	$C_{53}H_{73}N_{12}O_{13}S_2$	[1]
quinomycin monosulfoxide [a]	244; 210; 326	1117; $[M+H]^+$<br>1139; $[M+Na]^+$	$C_{51}H_{64}N_{12}O_{13}S_2$	[1;2]
Quinomycin Bo [a]	244; 210; 326	1129; $[M+H]^+$	$C_{53}H_{68}N_{12}O_{12}S_2$	[3]
Quinomycin G [a]	245.2; 325.8	1101; $[M+H]^+$	$C_{51}H_{64}N_{12}O_{12}S_2$	[4]
Quinomycins H1 [a]	233; 241; 312; 355	1138; $[M+Na]^+$	$C_{52}H_{65}N_{11}O_{13}S_2$	[5]
Quinomycins H2 [a]	233; 241; 312; 355	1138; [M+Na]+	$C_{52}H_{65}N_{11}O_{13}S_2$	[5]
QN-quinomycin A [b]	240; 292; 313-316	1083; $[M+H]^+$	$C_{52}H_{62}N_{10}O_{12}S_2$	[6]
NX-quinomycin A [b]	240; 317	1084; [M+H]	$C_{51}H_{61}N_{11}O_{12}S_2$	[6]
RK-1355A [a]	218; 230; 298; 356	1143; $[M+H]^+$	$C_{53}H_{62}N_{10}O_{15}S_2$	[7]
RK-1355B [a]	218; 230; 298; 356	1157; $[M+H]^+$	$C_{54}H_{64}N_{10}O_{15}S_2$	[7]
SW-163C [a]	214; 230; 299; 359	1113; $[M+H]^+$	$C_{52}H_{60}N_{10}O_{14}S_2$	[8]
SW-163E [a]	214; 230; 299; 359	1141; $[M+H]^+$	$C_{54}H_{64}N_{10}O_{14}S_2$	[8]
UK-63;598(SW-163D) [a]	218; 231; 299; 357	1127; $[M+H]^+$	$C_{53}H_{62}N_{10}O_{14}S_2$	[7;9]
UK-63;052(SW-163G) [a]	219; 231; 300; 359	1169; $[M+H]^+$	$C_{56}H_{68}N_{10}O_{14}S_2$	[9]
UK-65;662(SW-163F) [a]	219; 230; 300; 359	1155; $[M+H]^+$	$C_{55}H_{66}N_{10}O_{14}S_2$	[9]
triostin A [a]	243; 316; 326	1086; M^+	$C_{50}H_{62}N_{12}O_{12}S_2$	[10]
triostin C [a]	243; 315-326;	1142; M^+	$C_{54}H_{70}N_{12}O_{12}S_2$	[11]
Bis-6-chlorotriostin A [b]	335	1154; M^+	$C_{50}H_{60}N_{12}O_{12}S_2Cl_2$	[12]
Bis-6-bromotriostin A [b]	335	1242;M^+	$C_{50}H_{60}N_{12}O_{12}S_2Br_2$	[12]
Bis-7-chlorotriostin A [b]	335	1154; M+	$C_{50}H_{60}N_{12}O_{12}S_2Cl_2$	[12]
Bis-3-aminotriostin A [b]	312; 403	1116; M+	$C_{50}H_{64}N_{14}O_{12}S_2$	[12]
BBM-928 A (luzopeptin A) [a]	235; 264; 345	1426; M+	$C_{64}H_{78}N_{14}O_{24}$	[13]
BBM-928 B (luzopeptin B) [a]	235; 264; 345	1384; M+	$C_{62}H_{76}N_{14}O_{23}$	[13]
BBM-928 C (luzopeptin C) [a]	235; 264; 345	1342; M+	$C_{60}H_{74}N_{14}O_{22}$	[13]
quinoxapeptin A [a]	235; 264; 345	1499; [M+Na]+	$C_{68}H_{84}N_{16}O_{22}$	[14]
quinoxapeptin B [a]	235; 264; 345	1459; [M+Na]+	$C_{65}H_{80}N_{16}O_{22}$	[14]
Sandramycin [a]	217; 229; 356	1221; [M+H]+	$C_{60}H_{76}N_{12}O_{16}$	[15]
quinaldopeptin [a]	214; 230; 299; 359	1243; $[M+H]^+$	$C_{62}H_{78}N_{14}O_{14}$	[16]
BE-22179 [a]	218; 226; 290; 360	1061; [M+H]+	$C_{46}H_{48}N_{10}O_{12}S_4$	[17]
thiocoraline [a]	218; 230; 298; 360	1157; [M+H]+	$C_{48}H_{56}N_{10}O_{12}S_6$	[18]
22'-deoxythiocoraline [a]	210; 230; 299; 360	1141; [M+H]+	$C_{48}H_{56}N_{10}O_{11}S_6$	[19]
12'-sulfoxythiocoraline [a]	209; 230; 299; 360	1173; [M+H]+	$C_{48}H_{57}N_{10}O_{13}S_6$	[19]

a: natural products; b: compounds by biosynthesis.

Table 6. Information of soil samples.

Samples	Characteristic of Soil	Location	Sampling Depth
Sample 1	Rhizosphere soil of *Aegiceras corniculatum*	N:21°51′50″ E:108°27′80″	10 cm under surface
Sample 2	Rhizosphere soil of *Sonneratia apetala*	N:21°51′30″ E:108°28′25″	10 cm under surface
Sample 3	Rhizosphere soil of *Sonneratia apetala*	N:21°51′60″ E:108°28′55″	10 cm under surface
Sample 4	Rhizosphere soil of *Aegiceras corniculatum*	N:21°51′50″ E:108°27′90″	10 cm under surface
Sample 5	Rhizosphere soil of *Aegiceras corniculatum*	N:21°51′50″ E:108°27′22″	10 cm under surface
Sample 6	Rhizosphere soil of *Zizania*	N:21°51′60″ E:108°28′55″	10 cm under surface

Sample 7	Rhizosphere soil of *Aegiceras corniculatum*	N:21°50′35″ E:108°36′48″	10 cm under surface
Sample 8	Rhizosphere soil of *Aegiceras corniculatum*	N:21°50′35″ E:108°36′51″	10 cm under surface

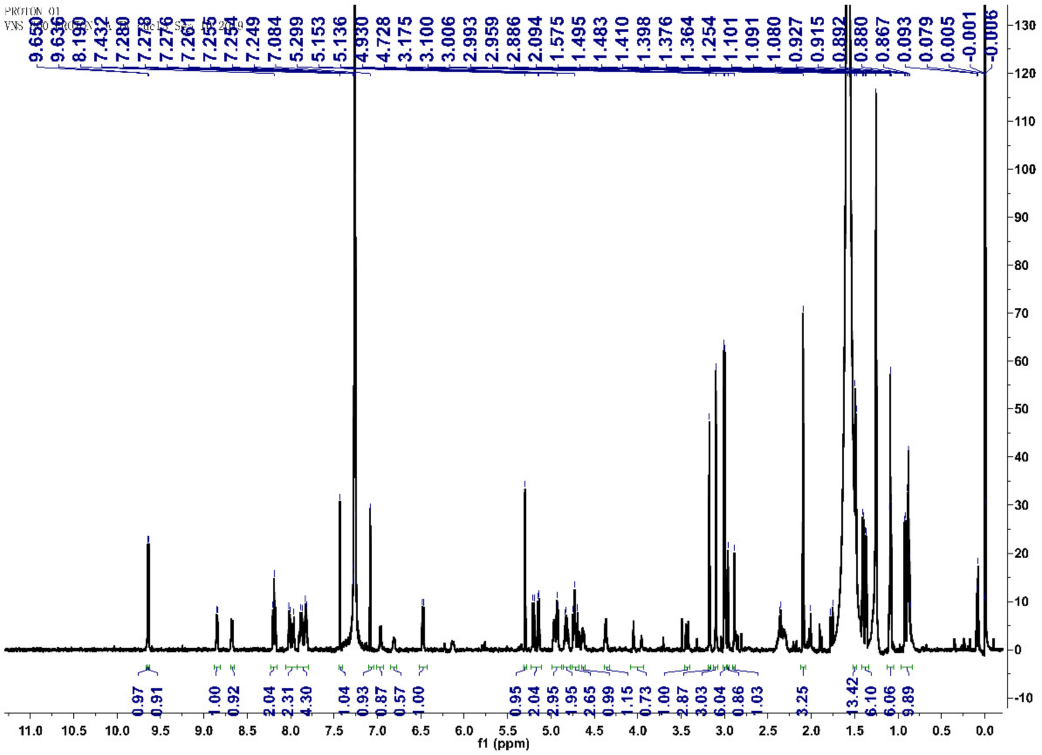

Figure 1. ^{1}H NMR (600 MHz) spectrum of peak **3** in $CDCl_3$.

A

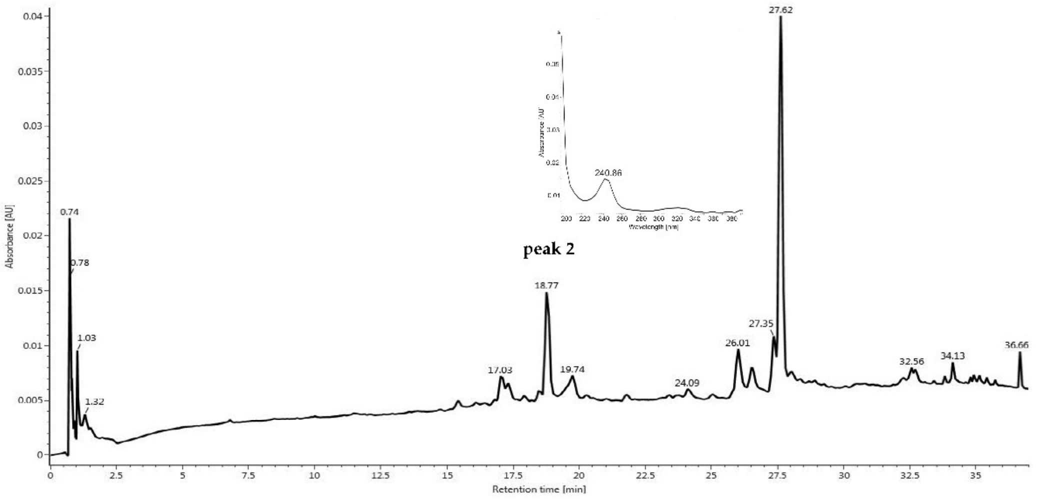

B

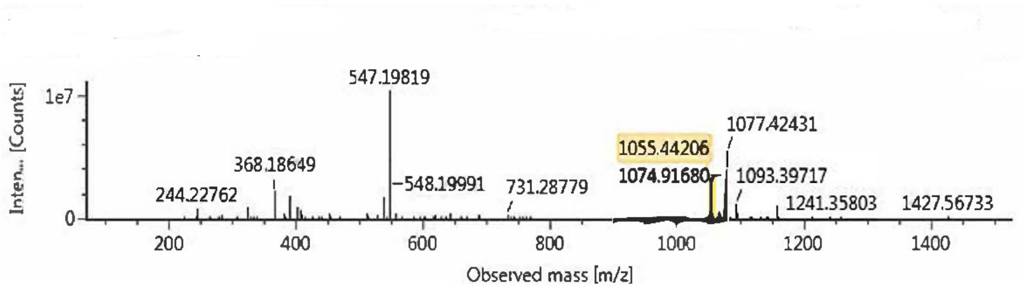

Figure 2. The UPLC-UV-HRMS/MS analysis and dereplication of peak **2**. (**A**) UPLC profile of peak **2** at Rt = 18.87 min and its UV spectra. (**B**) Mass spectra of peak **2**.

Reference List for Supplementary Material Table S5

1. Yang, Z.; Shao, L.; Wang, M.; Rao, M.; Ge, M.; Xu, Y. Two novel quinomycins discovered by UPLC-MS from *Stretomyces* sp. HCCB11876. *J. Antibiot.* **2019**, *72*, 164-168.
2. Huang, L.; XU, Y.W.; Kuang, Y.W.; Zhang, Q.L.; Wu Z.Z. Purification and Identification of Antitumor Secondary Metabolites from Soil *Streptomyces* sp. 2215. *Nat. Prod. Res. Dev.* **2009**, *21*, 235-238.
3. Shoji, J.; Konaka, R.; Kawano, K.; Higuchi, N.; Kyogoku, Y. Presence of isomers in quinomycin E. *J. Antibiot.* **1976**, *29*, 1246-1248.
4. Zhen, X.; Gong, T.; Liu F; Zhang, P.C.; Zhou, W.Q.; Li, Y.; Zhu, p. A New Analogue of Echinomycin and a New Cyclic Dipeptide from a Marine-Derived *Streptomyces* sp. LS298. *Mar. Drugs* **2015**, *13*, 6947-6961.
5. Hayakawa, Y.; Sone, R.; Aoki, H.; Kimata, S. Quinomycins H1 and H2, new cytotoxic antibiotics from *Streptomyces* sp. RAL404. *J. Antibiot.* **2018**, *71*, 898-901.
6. Yoshida, T.; Kimura, Y.; Katagiri, K. Novel quinomycins: Biosynthetic replacement of the chromophores. *J. Antibiot.* **1968**, *21*, 465-467.
7. Lim, C.L.; Nogawa, T.; Uramoto, M.; Okano, A.; Hongo, Y.; Nakamura, T.; Koshino, H.; Takahashi, S.; Ibrahim, D.; Osada, H. RK-1355A and B, novel quinomycin derivatives isolated from a microbial metabolites fraction library based on NPPlot screening. *J. Antibiot.* **2014**, *67*, 323-329.
8. Takahashi, K.; Koshino, H.; Esumi, Y.; Tsuda, E.; Kurosawa, K. SW-163C and E, Novel Antitumor Depsipeptides Produced by *Streptomyces* sp. *J. Antibiot.* **2001**, *54*, 622-627.
9. Rance, M.J.; Ruddock, J.C.; Pacey, M.S.; Cullen, W.P.; Huang, L.H.; Jefferson, M.T.; Whipple, E.B.; Maeda, H.; Tone, J. UK-63,052 complex, new quinomycin antibiotics from *Streptomyces braegensis* subsp. *japonicus*; taxonomy, fermentation, isolation, characterisation and antimicrobial activity. *J. Antibiot.* **1989**, *42*, 206-217.
10. Shin, M.; Inouye, K.; Otsuka, H. Synthetic Studies on Quinoxaline Antibiotics. II. Synthesis of Triostin A. *Bull. Chem. Soc. Jpn.* **1984**, *57*, 2203-2210.
11. Otsuka, H.; Shōji, J. The structure of triostin C. *Tetrahedron* **1965**, *21*, 2931-2938.
12. Cornish, A.; Fox, K.R.; Waring, M.J. Preparation and DNA-binding properties of substituted triostin antibiotics. *Antimicrob. Agents Chemother.* **1983**, *23*, 221-231.
13. Konishi, M.; Ohkuma, H.; Sakai, F.; Tsuno, T.; Koshiyama, H.; Naito, T.; Kawaguchi, H. Structures of BBM-928 A, B, and C. Novel Antitumor Antibiotics from *Actinomadura luzonensis*. *J. Am. Chem. Soc.* **1981**, *103*, 1241-1243.
14. Boger, D.L.; Ledeboer, M.W.; Kume, M.; Jin, Q. Total Synthesis of Quinoxapeptin A–C: Establishment of Absolute Stereochemistry. *Angew. Chem. Int. Ed.* **1999**, *38*, 2424-2426.
15. Matson, J.A.; Bush, J.A. Sandramycin, a novel antitumor antibiotic produced by a *Nocardioides* sp. production, isolation, characterization and biological properties. *J. Antibiot.* **1989**, *42*, 1763-1767.
16. Toda, S.; Sugawara, K.; Nishiyama, Y.; Ohbayashi, M.; Ohkusa, N.; Yamamoto, H.; Konishi, M.; Oki, T. Quinaldopeptin, a novel antibiotic of the quinomycin family. *J. Antibiot.* **1990**, *43*, 796-808.
17. Okada, H.; Suzuki, H.; Yoshinari, T.; Arakawa, H.; Okura, A.; Suda, H.; Yamada, A.; Uemura, D. A new topoisomerase II inhibitor, BE-22179, produced by a *Streptomycete*. I. Producing strain, fermentation, isolation and biological activity. *J. Antibiot.* **1994**, *47*,129-135.
18. Perez, B.J.; Canedo, L.M.; Puentes, J.L.F.; Elipe, M.V.S. Thiocoraline, a novel depsipeptide with antitumor activity produced by a marine *Micromonospora*. II. Physico-chemical properties and structure determination. *J. Antibiot.* **1997**, *50*, 738-741.
19. Wyche, T.P.; Hou, Y.; Braun, D.; Cohen, H.C.; Xiong, M.P.; Bugni, T.S. First Natural Analogs of the Cytotoxic Thiodepsipeptide Thiocoraline A from a Marine *Verrucosispora* sp. *J. Org. Chem.* **2011**, *76*, 6542-6547.

© 2019 by the authors. Licensee MDPI, Basel, Switzerland. This article is an open access article distributed under the terms and conditions of the Creative Commons Attribution (CC BY) license (http://creativecommons.org/licenses/by/4.0/).
